# An unusually large myofibroblastoma in a male breast: a case report

**DOI:** 10.1186/1752-1947-2-157

**Published:** 2008-05-14

**Authors:** Abeywardana MS Abeysekara, HP Priyantha Siriwardana, K F Abbas, Peter Tanner, Akin A Ojo

**Affiliations:** 1 Department of Surgery, King George Hospital, Barley Lane, Goodmayes, Essex IG3 8YB, UK; 2 Department of Pathology, King George Hospital, Barley Lane, Goodmayes, Essex IG3 8YB, UK

## Abstract

**Introduction:**

Myofibroblastoma of the breast is a rare benign stromal tumour seen predominantly in men. The gross appearance is that of a well-circumscribed nodule, characteristically small, seldom exceeding 3 cm. We present a case of an unusually large myofibroblastoma, which mimicked a malignant breast tumour.

**Case presentation:**

A 65-year-old man presented with a rapid enlargement of the right breast over 6 weeks. Examination revealed a firm 15 cm hemispherical lump occupying the whole of the right breast with peau d'orange appearance of the overlying skin and distortion of the nipple. The clinical and radiological features suggested the possibility of sarcoma of the breast. However, a guided Tru-Cut biopsy was inconclusive. A mastectomy was performed to remove the tumour, which weighed more than 2 kg. Histopathology and immunocytochemistry revealed a mixed classical and collagenised type of myofibroblastoma. The patient is well with no evidence of recurrence 5 years after the mastectomy.

**Conclusion:**

This unexpected presentation of an unusually large myofibroblastoma in a male breast is the largest reported to date. Myofibroblastomas can mimic malignant neoplasms and the clinical significance of this entity lies primarily in its recognition as a distinctive benign neoplasm.

## Introduction

Myofibroblastoma is a rare benign tumour of the breast predominantly seen in men in their sixth to seventh decades. It was first described by Wargotz et al. in 1987 [1]. Since then, more than 80 cases have been reported and their gross appearance is that of a well-circumscribed nodule, characteristically small, seldom exceeding 3 cm [2]. They are mesenchymal in origin and may exhibit a wide spectrum of histological features and a varied cellularity that can be misinterpreted as a sarcoma. We report a case of an unexpected presentation of a very large myofibroblastoma of a male breast, which mimicked malignant features clinically and radiologically.

## Case presentation

**Figure 1:**
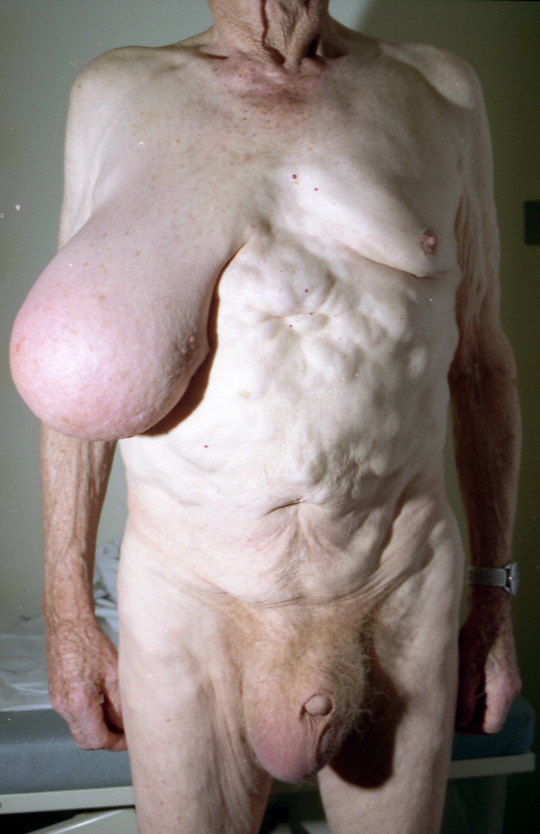
Photograph illustrating an enlarged right breast, due to myofibroblastoma, with nipple deviation, multiple lipomatosis on the trunk and a large hydrocele.

A 65-year-old man presented with swelling of his right breast of 6 weeks duration. The swelling was insidious at onset but increased rapidly in size. There was no history of pain or fever associated with the swelling. He was known to have familial lipomatosis with multiple other swellings on his body which had been present for several years. Examination revealed a non-tender, firm 15 cm hemispherical lump occupying the whole of the right breast with peau d'orange appearance of the overlying skin and distortion of the nipple. The left breast was normal. There was no palpable axillary lymphadenopathy. There were multiple non-tender, soft solid subcutaneous lumps (lipomas) in the trunk and there was also a large right sided hydrocele (Fig. 1). Based on the clinical findings of the breast swelling, it was thought to be a malignant tumour.

Routine baseline investigations, including chest X-ray, were within normal limits. Ultrasound scan of the breast revealed that it was an encapsulated mass containing areas of decreased echo pattern suggestive of sarcomatous changes in a lipoma. Magnetic resonance imaging of the breast swelling detected mixed signals with some areas of enhancement similar to those seen in soft tissue sarcomas. Computerised tomography ruled out any pulmonary metastasis. Mammography showed an enlarged dense right breast with a well-circumscribed soft tissue mass occupying almost the whole of the breast leaving a rim of normal breast tissue. Image guided core biopsy was inconclusive and the histological appearances were compatible with a wide differential diagnosis including fibromatosis, fasciitis, myofibroblastoma, dermatofibrosarcoma protuberance, leiomyoma or peripheral nerve sheath tumour. The patient underwent a mastectomy of the right breast. The mastectomy specimen contained a greatly enlarged male breast of 22 × 16 × 15 cm^3^, weighing 2255 g. Slicing of the specimen revealed a spherical, soft, degenerated 15 cm mass occupying almost the entire specimen. The cut surface of the tumour varied in appearance, mostly brown and soft with a firm white area. The tumour was well circumscribed and could be 'shelled out' from the surrounding breast tissue.

**Figure 2:**
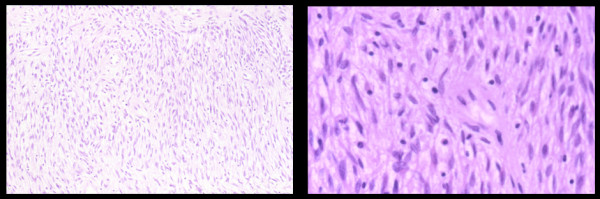
**A representative microscopic picture of a section of the myofibroblastoma.** This is a high-power view on the right side of the section.

Microscopic sections (Figure 2) revealed a tumour arising from breast connective tissue with areas which varied in cellularity. The cellular areas consisted of closely packed small uniform spindle cells showing relatively little dysplasia or pleomorphism and separated by varying amounts of collagen. Elsewhere myxoid degeneration was present. The white area noted macroscopically was of low cellularity, consisting of hyalinised collagen, which widely separated the tumour cells. The cells in this area showed greater pleomorphism and atypia. Mitotic figures were infrequent with no atypical mitoses seen. Although there was degeneration, no true necrosis was seen.

The tumour was extremely well circumscribed and had no infiltration to the adjacent breast tissue. The resection margin was free of tumour. Immunocytochemistry revealed that it was strongly positive for connective tissue markers vimentin and CD 34 and smooth muscle cell marker desmin, and negative for smooth muscle actin, protein S-100 (a marker for tumours of neural and fat origin) and epithelial marker MNF 116. Based on the histopathology and immunocytochemistry analysis, it was diagnosed as a myofibroblastoma of the breast. The stains for oestrogen and androgen receptors were not carried out. Following mastectomy, the patient made an uneventful recovery and, 5 years later, he remains well with no evidence of recurrence.

## Discussion

Soft tissue neoplasms of the breast that are composed of myofibroblasts have been classified as myofibroblastomas [1]. Myofibroblasts are spindle-shaped or fusiform mesenchymal cells derived from fibroblasts and are present in small numbers in all tissues. Proliferation of myofibroblasts is seen in various conditions including inflammatory reactions, fibromatosis and some sarcomas [3]. Ultrastructurally, myofibroblasts have features resembling myo-epithelial cells. Myofibroblasts are distinguished from spindle myo-epithelial cells largely on the basis of their distribution, immunohistochemical staining and electron microscopic characteristics [4]. Depending on their phenotypic state, both these types of cells may be reactive with anti-actin antibodies. Myoepithelial cells are typically positive for protein S-100 and cytokeratin in their epithelial phenotype, but myofibroblasts are negative. Their most common immunoprofile is diffuse desmin and CD 34 positivity [5]. Histological features and immunohistocytochemical features in this case were those of a myofibroblastoma. Microscopically, myofibroblastomas can be divided into five sub-types; classical, epitheloid, collagenised, cellular and infiltrative. In this case, the myofibroblastoma had features of mixed classical and collagenised type.

## Conclusion

Myofibroblastoma is a rare benign tumour of the breast. The tumour described in this report is unusual owing to its presentation, with very rapid enlargement mimicking a malignant tumour, and its large size, much greater than any previously reported. Malignant neoplasms, such as stromal sarcoma, malignant fibrous histiocytoma and spindle-cell sarcoma, or metaplastic carcinoma should not be confused with a myofibroblastoma. The clinical significance of this entity lies primarily in its recognition as a distinctive benign neoplasm.

## Competing interests

The authors declare that they have no competing interests.

## Authors' contributions

AMSA is the principal author of the paper, HPPS contributed in designing the paper and writing the introduction and discussion, KFA collected the data and contributed to the case presentation, PT revised and edited the histopathology description and discussion and AAO supervised the project and undertook the final revision before submission. All authors read and approved the final manuscript.

## Consent

Written informed consent was obtained from the patient for publication of this case report and any accompanying images. A copy of the written consent is available for review by the Editor-in-Chief of this journal.
